# Dietary Fructose Increases the Sensitivity of Proximal Tubules to Angiotensin II in Rats Fed High-Salt Diets

**DOI:** 10.3390/nu10091244

**Published:** 2018-09-06

**Authors:** Agustin Gonzalez-Vicente, Nancy J. Hong, Nianxin Yang, Pablo D. Cabral, Jessica M. Berthiaume, Fernando P. Dominici, Jeffrey L. Garvin

**Affiliations:** 1 Department of Physiology and Biophysics, School of Medicine, Case Western Reserve University, Cleveland, OH 44106, USA; nxh156@case.edu (N.J.H.); nxy66@case.edu (N.Y.); pdcabral@gmail.com (P.D.C.); jessica.berthiaume@case.edu (J.M.B.); jlg5@case.edu (J.L.G.); 2 Facultad de Farmacia y Bioquímica, Universidad de Buenos Aires, Ciudad Autónoma de Buenos Aires C1113AAD, Argentina; dominici@qb.ffyb.uba.ar; 3 Facultad de Medicina, Departamento de Ciencias Fisiológicas, Universidad de Buenos Aires, Ciudad Autónoma de Buenos Aires C1121ABG, Argentina; 4 Merk Research Laboratories, 630 Gateway Blvd, South San Francisco, CA 94080, USA; 5 Instituto de Química y Fisicoquímica Biológicas, Consejo Nacional de Investigaciones Científicas y Técnicas (CONICET), Ciudad Autónoma de Buenos Aires C1113AAD, Argentina

**Keywords:** kidney, blood pressure, salt sensitivity, Na^+^/H^+^ exchange, Na^+^/K^+^-ATPase, atrial natriuretic peptide, norepinephrine, sodium excretion

## Abstract

Dietary fructose causes salt-sensitive hypertension. Proximal tubules (PTs) reabsorb 70% of the filtered NaCl. Angiotensin II (Ang II), atrial natriuretic peptide (ANP) and norepinephrine (NE) regulate this process. Although Ang II signaling blockade ameliorates fructose-induced salt-sensitive hypertension, basal PT Na^+^ reabsorption and its sensitivity to the aforementioned factors have not been studied in this model. We hypothesized consuming fructose with a high-salt diet selectively enhances the sensitivity of PT transport to Ang II. We investigated the effects of Ang II, ANP and NE on PT Na reabsorption in rats fed a high-salt diet drinking tap water (HS) or 20% fructose (HS-FRU). Oxygen consumption (QO_2_) was used as a measure of all ATP-dependent transport processes. Na^+^/K^+^-ATPase and Na^+^/H^+^-exchange (NHE) activities were studied because they represent primary apical and basolateral transporters in this segment. The effect of 10^−12^ mol/L Ang II in QO_2_ by PTs from HS-FRU was larger than HS (*p* < 0.02; *n* = 7). In PTs from HS-FRU 10^−12^ mol/L Ang II stimulated NHE activity by 2.6 ± 0.7 arbitrary fluorescence units/s (*p* < 0.01; *n* = 5) but not in those from HS. The stimulatory effect of Ang II on PT Na^+^/K^+^-ATPase activity was not affected by HS-FRU. Responses of QO_2_ and NHE activity to ANP did not differ between groups. The response of QO_2_ to NE was unaltered by HS-FRU. We concluded that the sensitivity of PT Na^+^ reabsorption specifically to Ang II is enhanced by HS-FRU. This maintains high rates of transport even in the presence of low concentrations of the peptide, and likely contributes to the hypertension.

## 1. Introduction

Globalization and economic growth made processed foods available throughout the world [1]. Modern diets are particularly rich in NaCl and in fructose or fructose-containing syrups used as sweeteners [1]. Elevated Na^+^- and fructose-intake are detrimental to human health and are a public concern. The global consumption of Na^+^ is about 4 g/day/person [2], with values up to 30% higher than the world average in certain regions of Asia [2,3]. In North America and Europe, the average is near the world mean [2,4,5], while sub-Saharan Africa and South America present values close to 3 g/day/person [2]. All values are substantially higher than the upper safe limit by the World Health Organization and the American Heart Association, which are close to 2 g of Na^+^/person/day [3,6,7].

The addition of low-cost high-fructose corn syrup to processed foods and sugar-sweetened beverages raised the average calories ingested from fructose above 10% of the recommended caloric intake [8,9,10]. Unlike glucose or sucrose [11], acute ingestion of fructose elevates blood pressure and peripheral resistance [11,12,13]. In addition, cross sectional studies using questioners to estimate fructose intake, associate elevated chronic consumption of this sugar with hyperuricemia [14], insulin resistance [15] and increased cardiovascular risk [16,17]. In particular, those individuals consuming more than 74 g/day of fructose, equivalent to about 15% caloric intake on a 2000 kcal/day diet, present higher blood pressure values [17]. Finally, consumption of sugar-sweetened beverages has been associated with obesity in children [18], elevated uric acid and blood pressure in adolescents [19], and type II diabetes in adults [20]. In Asian countries such as China consumption of sugar-sweetened beverages has doubled versus the previous decade [21,22].

While chronic interventional studies are difficult to conduct in humans, rodent models offer a tool to study the interaction between fructose and Na^+^ consumption on cardiovascular health. As such, diets in which the Na^+^ content is near 16 g/kg as oppose to a 2.5 g Na^+^/kg of a regular rodent chow cause salt-sensitive hypertension in both rats [23,24] and mice [25]. Interestingly, Na^+^ restricted diets ameliorate hypertension even in very aggressive models such those giving 40% [26] or 66% [27] fructose by weight in the food.

The majority of the Na^+^ filtered by the glomerulus is reabsorbed by proximal tubules. The bulk of Na^+^ enters proximal tubule cells in exchange for intracellular protons via Na^+^/H^+^ exchangers (NHEs) located in the brush-border; the remainder energizes specific cotransporters including Na^+^/glucose-linked transporters 1, 2, 4 and 5, Na^+^/phosphate cotransporters, Na^+^/amino acid cotransporters and others. The gradient for Na^+^ entry via these processes is generated by the Na^+^/K^+^-ATPase located in the basolateral membrane.

Angiotensin II (Ang II), atrial natriuretic peptide (ANP) and norepinephrine (NE) regulate proximal nephron fluid and Na^+^ reabsorption. Ang II is one of the primary stimulators of proximal tubule Na^+^ reabsorption as demonstrated by the fact that augmenting and inhibiting Ang II signaling specifically in the proximal tubule increases and decreases blood pressure, respectively [28,29]. Sub-nanomolar Ang II concentrations stimulate both Na^+^ entry [30] via NHEs and exit via Na^+^/K^+^-ATPase [31]. Interfering with the effects of Ang II blunts the increase in blood pressure in fructose-fed rats [32,33,34,35,36,37]. Furthermore, we reported that dietary fructose in the absence of a high-salt diet enhances the sensitivity of proximal nephron Na^+^ transport to the stimulatory effect of Ang II [38]. Together these data support a role for both Ang II and proximal tubules in salt-sensitive hypertension induced by fructose but this has not been studied.

When blood pressure increases, pressure natriuresis eventually increases Na^+^ excretion establishing a new steady state [39]. These mechanisms are not well understood but have been reported to involve the proximal tubule [40], in addition to other nephron segments [41,42]. Currently it is not known whether pressure natriuretic mechanisms, as would be activated by salt-sensitive hypertension induced by fructose, mitigate the enhanced sensitivity of proximal nephron Na^+^ reabsorption to Ang II caused by fructose.

In contrast to Ang II, atrial natriuretic peptide (ANP) is a major inhibitory hormone of proximal nephron transport [43,44]. Elevated plasma ANP stimulates urinary Na^+^ excretion during volume expansion [45], such as likely occurs during fructose-induced salt sensitive hypertension, and it enhances urinary Na^+^ excretion in part through actions on the proximal tubule [46]. Activation of protein kinase C by Ang II blunts the effects of ANP [47,48] and acute fructose treatment enhances the effects of Ang II via protein kinase C when rats are fed a low-salt diet [49]. Given that dietary fructose sensitizes proximal tubule Na^+^ reabsorption to Ang II [38], one might expect that the effect of ANP on proximal tubule transport would be blunted by dietary fructose when rats are on a high-salt diet. However, it is unknown whether the response of proximal tubules to ANP is altered during fructose-induced salt-sensitive hypertension.

The proximal nephron is richly innervated with sympathetic nerves that stimulate Na^+^ reabsorption via the release of NE [50]. Blunting the actions of NE reduces the detrimental renal effects caused by dietary fructose [51]. Currently it is unknown whether such diets enhance the sensitivity of proximal nephron Na^+^ reabsorption to NE similar to Ang II.

Our hypothesis was that consuming fructose with a high-salt diet selectively enhances the sensitivity of proximal tubule transport to Ang II.

## 2. Materials and Methods

Drugs and buffers: Unless specified, all drugs and reagents were obtained from Sigma-Aldrich (St. Louis, MO, USA). The compositions of all solutions used in this study are shown in Table 1.

Bicarbonate-buffered physiological saline was continuously gassed with 95% O_2_/5% CO_2_ at 37 °C. For 4-(2-hydroxyethyl)-1-piperazineethanesulfonic acid (HEPES)-buffered physiological saline, acid pulse buffer and K^+^-free HEPES-buffered solution, the pH was titrated to 7.5 with NaOH and osmolality to 300 mOsmol/L with mannitol. The 4× reaction media, and the 4× reaction media with ouabain contained 6 U/mL pyruvate kinase (PK) and 9 U/mL lactic dehydrogenase (LDH). The PK/LDH enzyme mix (P0294, Sigma, St. Louis, MO, USA,) was also the source of 1 mmol/L KCl. Both solutions contained 0.1% dimethyl sulfoxide (DMSO) as an ouabain carrier. The pH-sensitive fluorescent dye 2′,7′-bis-(2-carboxyethyl)-5-(and-6)-carboxyfluorescein acetoxymethyl ester (BCECF-AM; Molecular Probes-Eugene, OR, USA) was dissolved daily in anhydrous DMSO and diluted in HEPES-buffered physiological saline to reach a final concentration of 1 μmol/L.

Animals: This study was approved by the Case Western Reserve University Institutional Animal Care and Use Committee. All experiments were conducted in accordance with the National Institutes of Health Guidelines for the Care and Use of Laboratory Animals. Six week-old male Sprague-Dawley rats (Charles River Breeding Laboratories, Wilmington, MA, USA) were used. 

**Dietary Treatment:** After arrival at our animal facility, all rats were fed a purified diet (Test-Diet #9GDV; St. Louis, MO, USA). This diet was designed to match the components of Test-Diet #5876 used in our previous study [38] with the exception of having 700 mEq Na^+^/kg (~16 g of Na^+^/kg) instead of 100 mEq Na^+^/kg. Rats were randomly assigned to two experimental groups: one receiving tap water; and the other drinking 20% fructose. These groups were called HS and HS-FRU respectively. The fructose solution was replaced every other day to prevent contamination.

**Protocol 1:** To measure blood pressure and blood chemistry, 12 animals were randomly assigned to either HS or HS-FRU on day 0. Three animals were housed per cage. Animals were trained on days 1, 3 and 5 for tail-cuff plethysmography. On day 7 blood pressures were measured as we previously described [52,53]. Blood was obtained from the tail with a lancet and glucose measured with a glucometer (FreeStyle Precision Neo^®^, Abbott Diabetes, Oxfordshire, UK). On Day 8, animals were anesthetized with isoflurane using 100% O_2_ as a carrier, and blood was drawn from the thoracic aorta. An aliquot of blood was used to measure pH, Na^+^, K^+^, Cl^−^ and lactate using a Nova Prime Blood Analyzer (Nova Biomedicals, Walthman, MA, USA). The remainder was used to collect plasma. Insulin was determined on frozen plasma samples using a Rat Insulin enzyme-linked immunosorbent assay (ELISA) kit (MERCODIA AB, Uppsala, Sweden) according to manufacturer recommendations.

**Protocol 2:** To measure food and fluid consumption and growth, 12 animals were housed individually and weighed (day 0). Then they were randomly assigned to either HS or HS-FRU. On day 5, 6 and 7, the animals were weighed, and food and fluid intake measured. We reported the average food and fluid intake and weight gain for those days. These data were used to calculate the daily caloric and Na^+^ intakes, and the percentage of calories from fructose.

**Protocol 3:** For each experiment requiring terminal surgery, a different set of animals was used. The number of animals used for each experiment is mentioned in the results section. Animals were housed in pairs and assigned to either HS or HS-FRU for 7 to 9 days. Rats were anesthetized with ketamine (100 mg/kg bw) and xylazine (20 mg/kg bw) administered by intraperitoneal injection. Only one sample, either a proximal tubule suspension or a microdissected proximal tubule was obtained per animal.

Proximal tubule suspension: proximal tubule suspensions were generated using methods similar to those we used before [38]. Briefly, rats were anesthetized and received 2 units of heparin by intraperitoneal injection. Kidneys were digested in situ by perfusing them at 0.7 mL/min with 80 mL of bicarbonate-buffered physiological saline containing 1 mg/mL collagenase and 2 IU/mL heparin, at 37 °C. Once digested, kidneys were excised and immediately cooled by submersion in bicarbonate-buffered physiological saline at 4 °C. Kidney cortexes were gently scraped with a blade, minced, and transferred to a 5 mL conical tube. Proximal tubules were loosened from the cortical digest by passing it through a wide orifice pipette tip and stirring on ice for 5 min. The resultant suspension was filtered through a 390 µm mesh, and the tubules recovered by centrifugation at 4 °C (100× *g* for 2 min). The tubules were rinsed, filtered again through a 250 µm mesh, and recovered by centrifugation at 4 °C (100× *g* for 2 min). The final pellet was resuspended in 5 to 10 mL of warm gassed bicarbonate-buffered physiological saline. After sitting for 1 min to sediment glomeruli, 3 mL from the upper portion were taken for experiments.

Oxygen consumption (QO_2_): changes in QO_2_ by proximal tubules are a surrogate for changes in net transport rates because this segment produces ATP via aerobic metabolism, and the majority of this ATP (60–70%) is used to drive Na^+^ across the basolateral membrane by the Na^+^/K^+^-ATPase. QO_2_ was measured using methods similar to those we reported [38,54]. Briefly, 2 to 4 mg of protein suspended in bicarbonate-buffered physiological saline were taken to a final volume of 6 mL in the chamber of a YSI Model 5301B bath assembly (Yellow Springs Instruments, Yellow Springs, OH, USA). The chamber was equilibrated at 37 °C with a gas mix containing 95% O_2_ and 5% CO_2_ and then closed. The oxygen tension in the chamber was monitored using a YSI Model 5300 Biological Oxygen Monitor (Yellow Springs Instruments, Yellow Springs, OH, USA) attached to a PowerLab (ADInstruments, Colorado Springs, CO, USA). After stabilizing for 90 s, basal QO_2_ was recorded for 1 min. Then the effects of increasing concentrations of either Ang II, ANP or NE were assessed as indicated in the Results section. At the end of the experiment, tubules were recovered by centrifugation to determine protein content. The results were expressed as nmol O_2_/mg protein/min.

Proximal tubule perfusion: after rats were anesthetized the abdominal cavity was opened and the left kidney bathed in ice-cold 150 mmol/L NaCl. Immediately after, the kidney was excised and submerged in 50 mL of ice-cold HEPES-buffered physiological saline. The kidney was transferred to a cold Lucite plate and coronal slices cut from the midsection. Single S2 segments of proximal tubules were dissected from cortical slices free-hand on a stereomicroscope stage in HEPES-buffered physiological saline cooled to less than 10 °C. Segments ranging from 0.7 to 1.0 mm were transferred to a temperature-regulated chamber and microperfused using concentric glass pipettes as we have previously described [38].

Measurement of NHE activity: A 1 mmol/L stock solution of the pH-sensitive dye BCECF-AM was prepared fresh daily. Proximal tubules were bathed and perfused with HEPES-buffered physiological saline at 37 ± 1 °C, and loaded with dye by adding 1 μmol/L BCECF-AM in the basolateral bath for 5 min and then washing them for 10 min. BCECF was alternately excited at 490 and 450 nm. Emitted fluorescence was collected at 535 ± 25 nm using a 40× immersion oil objective and a Coolsnap HQ digital camera (Photometrics, Tucson, AZ, USA). Images were recorded and analyzed with Metafluor version 7 imaging software (Universal Imaging, Downingtown, PA, USA).

Initial fluorescence was measured for 1 min. Then, intracellular pH (pH_i_) was reduced using the ammonium pulse method as previously described [38,49]. The initial rate of pH_i_ recovery was taken as a measure of NHE activity. Data were collected at 2 s intervals, and NHE activity expressed as arbitrary fluorescence units per second (AFU/s). Each tubule was subjected to two periods, one basal and one with the study compound with a 10 min recovery and re-equilibration period in between measurements. Ang II or ANP were added to the basolateral bath.

Na^+^/K^+^-ATPase activity: the hydrolytic activity of Na^+^/K^+^-ATPase was measured by coupling ADP production to NAD^+^ generation as described previously [38]. Briefly, an aliquot of proximal tubule suspension was rinsed with K^+^-free HEPES-buffered solution and placed on a dissecting microscope. Using micro tweezers, 2 tubules totaling not less than 0.5 mm were transferred to a 0.5 mL “safe-lock”-Eppendorf tube containing 30 µL of 0.7% octylglucoside and either vehicle or Ang II. Tubules were incubated on ice for 10–15 min, and then 13 µL of 4× reaction media were added to all tubes except those used to measure ouabain-insensitive ATPase activity which received 4× reaction media with ouabain. Samples were incubated at 37 °C for 30 min. The reaction was stopped by adding 53 µL of 0.5 mol/L HCl to each tube and incubating the mix for 15 min at 37 °C. Finally, the whole reaction volume was transferred to a 2 mL Eppendorf tube containing 1 mL of 6 mol/L KOH and incubated at 60 °C for 20 min in the dark. All samples were measured on a Hitachi F2700 spectrofluorometer (Hitachi, Tokyo, Japan), exciting at 366 nm and collecting light at 455 nm. The assay was calibrated over a range of ADP from 0.5 to 3 nmoles. Na^+^/K^+^-ATPase hydrolytic activity was expressed as pmol ATP/mm/min.

Statistics: data were analyzed using GraphPad Prism V6.07 (GraphPad Inc., La Jolla, CA, USA) and R, version 3.2.3 (R Foundation for Statistical Computing, Vienna, Austria. URL https://www.R-project.org/). Results are expressed as the mean ± the standard error of the mean for each group, or the difference between two means ± the standard error of that difference. The standard error of the difference between means was calculated as follows: SE_ΔAB_ = sqrt(SD_A_^2^/n_A_ + SD_B_^2^/n_B_); where for each mean, A or B, SD is the standard deviation and n is the sample size. For comparison of two means unpaired 2-tailed Student *t*-test was used. Statistical analysis of data to assess the effects of diet alone, hormone alone, and their interactions was performed by two-way analysis of variance (ANOVA) for repeated measures. Hochsberg’s method was used to correct for multiplicity in post hoc testing. If no interaction was found, no post hoc testing was performed. All *p* values < 0.05 were considered significant. Corrected *p* values are reported as appropriate.

## 3. Results

To start studying this model we evaluated the nutritional profile and growth as presented in Table 2. Animals did not present differences in final weight or growth rate. Fluid consumption by the HS-FRU rats was less due to reduced food, and thus salt, intake. Although food intake was lower in HS-FRU, the difference in calories was made up by the calories consumed as fructose. As a result, total calories consumed by both groups were not significantly different. Data presented in Table 3 show that fructose feeding did not affect blood glucose and insulin levels, nor the acid–base balance as measured by lactate and pH. The main electrolytes Na^+^, K^+^ and Cl^−^ were within normal ranges, and there were no differences between groups.

**Figure 1:**
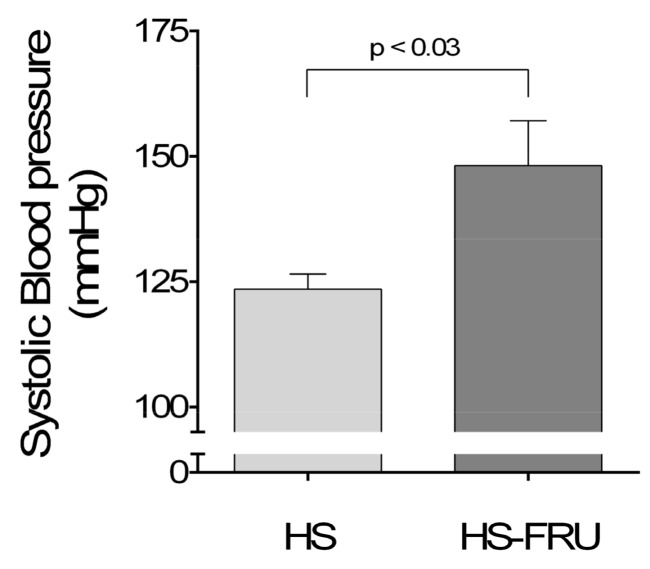
Effect of drinking 20% fructose for 7 days while on a high-salt diet on systolic blood pressure. Rats fed a high-salt diet drinking tap water (HS) or 20% fructose (HS-FRU).

To begin to test our hypothesis we measured the effect of dietary fructose on blood pressure while rats were on a high-salt base diet. We found that blood pressure in rats given fructose and high salt was 148 ± 9 mmHg (*n* = 6) as opposed to 123 ± 3 mmHg (*n* = 6) in animals fed high salt only (Δ + 25 ± 9 mmHg; *p* < 0.03; Figure 1).

**Figure 2:**
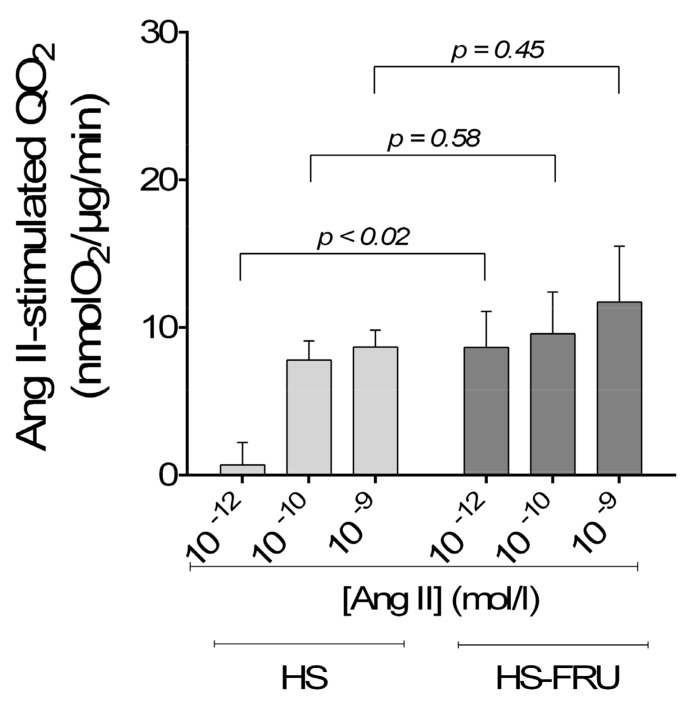
Effect of angiotensin II (Ang II) on oxygen consumption (QO_2_) by proximal tubule suspensions from rats fed a high-salt diet drinking tap water (HS) or 20% fructose (HS-FRU). *n* = 7 for each group. By two-way analysis of variance (ANOVA): Ang II effect (*p* < 0.001); fructose effect (*p* = 0.20); interaction (*p* < 0.02). *p* values from post-hoc testing are depicted in the figure.

Then we studied the effect of fructose on basal QO_2_ by proximal tubule suspensions. Basal rates of QO_2_ by proximal tubules from HS and HS-FRU were not significantly different as expected. Subsequently we explored the sensitivity of proximal tubule active transport to Ang II by investigating the effect of varying concentrations of Ang II on QO_2_ (Figure 2). The effect of Ang II alone (*p* < 0.001) and the interaction between Ang II and fructose (*p* < 0.02) were statistically significant when analyzed by two-way ANOVA. The effect of dietary fructose alone was not (*p* = 0.20). Post-hoc testing showed that 10^−12^ mmol/L Ang II significantly increased QO_2_ in tubules from the HS-FRU group compared to HS alone (*p* < 0.02; *n* = 7 for each group). The effect of Ang II at other concentrations was not statistically different between groups.

**Figure 3:**
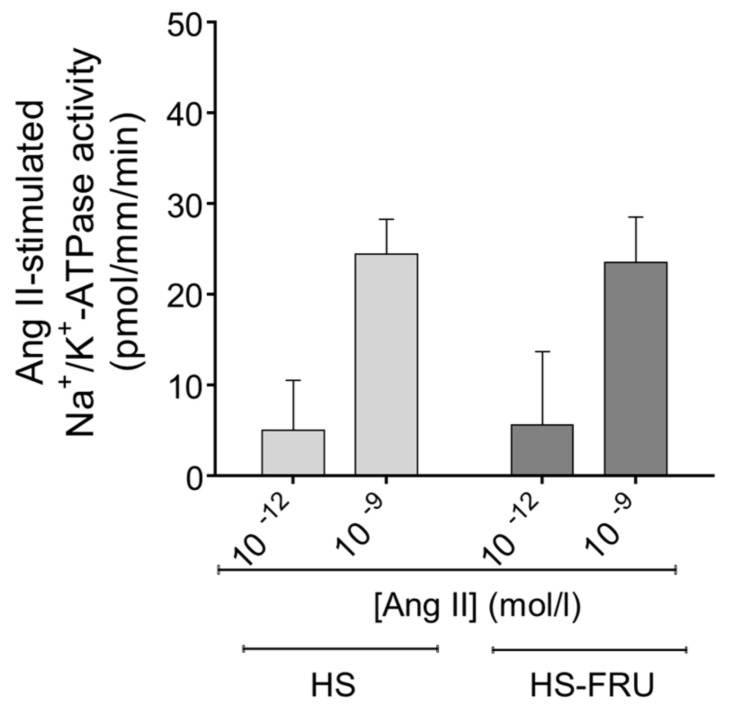
Effect of angiotensin II (Ang II) on Na^+^/K^+^-ATPase hydrolytic activity in proximal tubules from rats fed a high-salt diet drinking tap water (HS) or 20% fructose (HS-FRU). By two-way ANOVA: Ang II effect (*p* < 0.003); fructose effect (*p* = 0.98); interaction (*p* = 0.88).

Since all proximal tubule transport ultimately depends on Na^+^/K^+^-ATPase, we next studied the effects of Ang II (10^−12^ and 10^−9^ mol/L) on this transporter. Na^+^/K^+^-ATPase was assessed by the ATP hydrolytic activity of permeabilized tubules, where transmembrane Na^+^ gradients are negligible. Under these conditions, Ang II (10^−12^ mol/L) did not significantly enhance Na^+^/K^+^-ATPase activity in tubules from either group (Figure 3). In HS-FRU, Na^+^/K^+^-ATPase was 43 ± 6 pmol ATP/mm/min without Ang II and 49 ± 8 pmol ATP/mm/min with Ang II (*n* = 8). In HS, the values were 36 ± 4 pmol ATP/mm/min without Ang II and 41 ± 7 pmol ATP/mm/min with Ang II (*n* = 8). Ang II (10^−9^ mol/L), used as a positive control, stimulated Na^+^/K^+^-ATPase activity to the same extent in tubules from both groups (69 ± 7 pmol ATP/mm/min in HS-FRUC vs. 61 ± 7 pmol ATP/mm/min in HS). When the data were analyzed by two-way ANOVA, the only factor that was significant was the effect of Ang II (*p* < 0.003). Neither the effect of fructose alone nor the interaction was significant.

**Figure 4:**
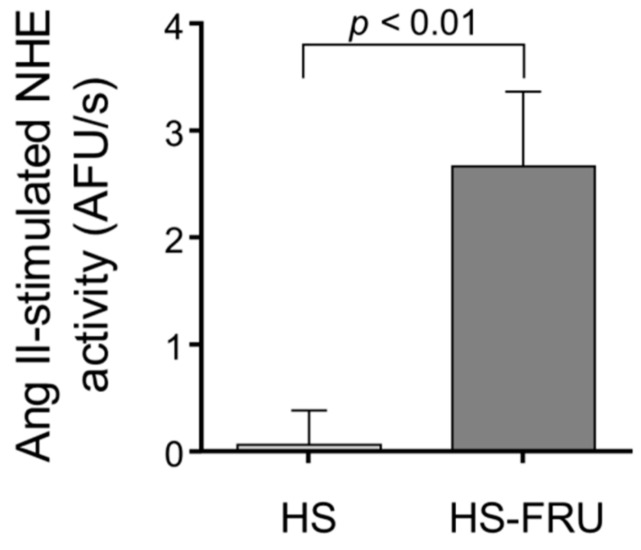
Effect of 10^−12^ mol/L angiotensin II Ang II on Na^+^/H^+^-exchanger (NHE) activity in proximal tubules isolated from rats fed a high-salt diet drinking tap water (HS) or 20% fructose (HS-FRU).

Because Na^+^ exit via Na^+^/K^+^-ATPase was unaffected by HS-FRU and NHE mediates the vast majority of Na^+^ entry into proximal tubules, we next measured NHE activity. In the HS-FRU tubules, 10^−12^ mol/L Ang II increased NHE activity by 2.7 ± 0.7 AFU/s, while the effect in HS was only 0.1 ± 0.3 AFU/s (difference between groups 2.6 ± 0.7 AFU/s; *p* < 0.01; *n* = 5 for each group; Figure 4). These results show that in a high-salt base diet, a low concentration of Ang II only stimulated NHE activity in tubules from rats receiving fructose in the drinking water.

**Figure 5:**
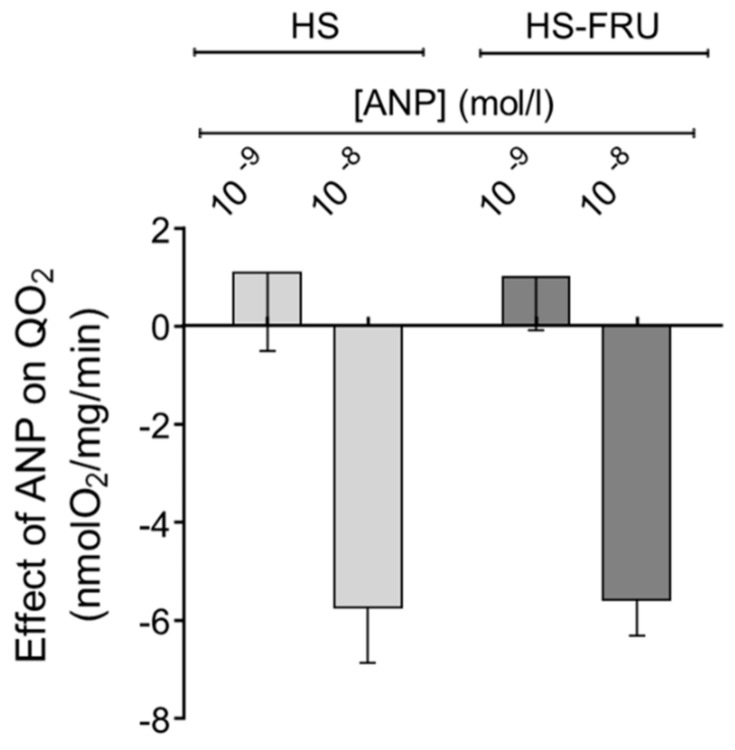
Effect of atrial natriuretic peptide (ANP) on oxygen consumption (QO_2_) by proximal tubule suspensions from rats fed a high-salt diet drinking tap water (HS) or 20% fructose (HS-FRU). By two-way ANOVA: ANP effect (*p* < 0.005); fructose effect (*p* = 0.99); interaction (*p* = 0.91).

It has been shown that under normal circumstances ANP plays a role in eliminating excess Na^+^ by decreasing proximal tubule transport, and this effect might be reduced in individuals with salt-sensitive hypertension. Therefore, we tested the effect of ANP on QO_2_ in proximal tubule suspensions. 10^−8^ mol/L ANP reduced QO_2_ by 5.6 ± 0.7 nmol O_2_/mg/min (*n* = 6) in HS-FRU and by 5.8 ± 1.0 nmol O_2_/mg/min in HS proximal tubules (Figure 5; *n* = 6). A lower concentration of the peptide (10^−9^ mol/L) had no significant effect on QO_2_ by either group. When analyzed by two-way ANOVA, the effect of ANP alone was statistically significant (*p* < 0.005) but neither the effect of fructose alone nor the interaction between fructose and ANP were.

**Figure 6:**
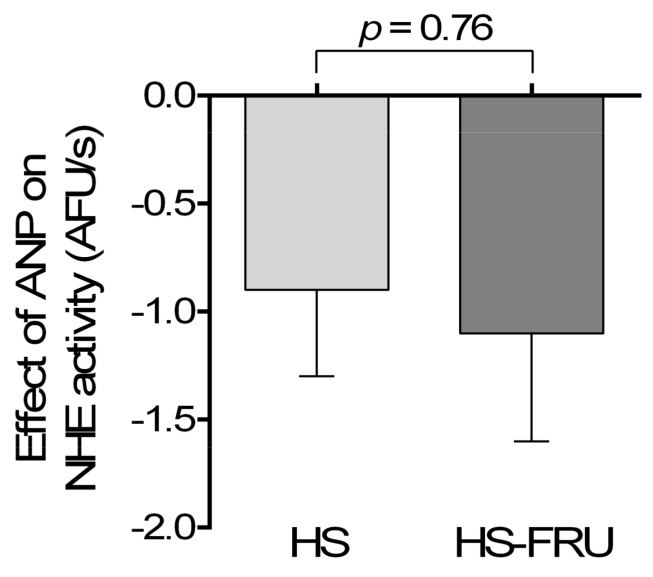
Effect of 10^−8^ mol/L atrial natriuretic peptide (ANP) on Na^+^/H^+^-exchanger (NHE) activity in proximal tubules isolated from rats fed a high-salt diet drinking tap water (HS) or 20% fructose (HS-FRU).

Similar to the experimental protocol for Ang II, we next tested the effects of ANP on NHE activity in proximal tubules from rats fed either HS or HS-FRU. Figure 6 shows that in HS-FRU 10^−8^ ANP reduced the rate of pH_i_ recovery from 2.1 ± 0.5 to 1.0 ± 0.4 AFU/s (Δ − 1.1 ± 0.5 AFU/s; *n* = 7). Similarly, in HS 10^−8^ ANP reduced the rate of pH_i_ recovery from 2.4 ± 0.3 to 1.5 ± 0.4 AFU/s (Δ − 0.9 ± 0.4 AFU/s; *n* = 5). These data suggest that the inhibitory effects of ANP on NHE activity are not affected by fructose when rats are on high salt.

**Figure 7:**
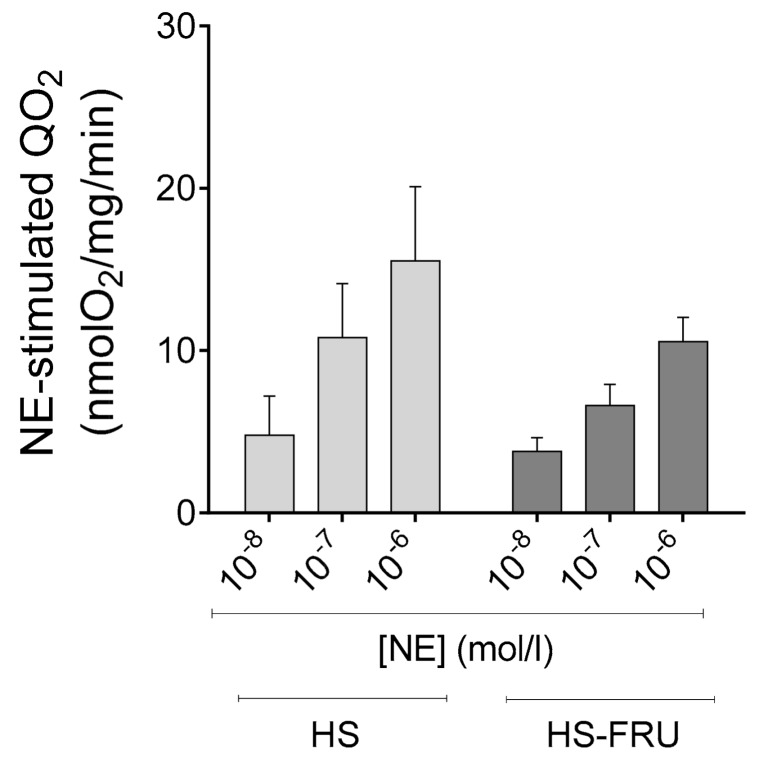
Effect of norepinephrine (NE) on oxygen consumption (QO_2_) by proximal tubule suspensions from rats fed a high-salt diet drinking tap water (HS) or 20% fructose (HS-FRU). By two-way ANOVA: NE effect (*p* < 0.03); fructose effect (*p* = 0.96); interaction (*p* = 0.54).

Finally, to test whether the effects of dietary fructose were specific for Ang II, we studied regulation of transport by the catecholamine NE. Three different concentrations of NE were used. Addition of 10^−8^, 10^−7^ and 10^−6^ mol/L NE stimulated QO_2_ by 4.8 ± 2.4, 10.8 ± 3.3 and 15.5 ± 4.6 nmol/mg/min in tubules from HS. For HS-FRU the values were 3.8 ± 0.8, 6.6 ± 1.3 and 10.6 ± 1.5 (Figure 7; *n* = 6 for each group). When analyzed by two-way ANOVA the effect of NE alone was statistically significant (*p* < 0.03) but the effect of fructose alone and the interaction were not. These data indicate that HS-FRU does not alter the response to NE. 

## 4. Discussion

Our hypothesis was that during fructose-induced salt-sensitive hypertension the sensitivity of the rat proximal tubule to Ang II is enhanced. We found that when animals are on a base diet containing 700 mEq/kg Na^+^, 200 g/Kg fructose in the drinking water: (1) increased blood pressure; (2) enhanced the ability of a low concentration of Ang II to stimulate QO_2_, which was due to a primary elevation of NHE activity rather than Na^+^/K^+^-ATPase; and (3) did not alter the response of the proximal nephron to ANP or NE. Thus, our data indicate that 20% fructose in the drinking water causes low concentrations of Ang II to stimulate net proximal tubule transport when animals are on a high-salt diet leading to hypertension.

We first tested the effect of a 20% fructose beverage on blood pressure. Previously we [38,49,55] and others [23,24] had shown that giving rats a 20% fructose solution as a sole source of fluid does not cause a significant increase in blood pressure when animals are on a base diet that contained 100 mEq Na^+^/kg (Regular rodent chow). It was also demonstrated that when rats eating regular rodent chow are given a 20% fructose beverage for 1 week, switching the Na^+^ content of the diet to 700 mEq Na^+^/kg elevates blood pressure [23,49,56]. The data presented here are the first showing that no fructose preconditioning or “priming” is necessary, and that starting the 20% fructose and high-salt dietary treatments simultaneously causes hypertension within one week.

The difference in blood pressures was not due to differences in starting weights, growth rates or weights at the end of the study. Although HS-FRU consumed less food, their caloric intake was the same as the HS group due to drinking 20% fructose.

Down-regulation of the renin-angiotensin system is an important mechanism to eliminate excess salt. When animals are given high-salt diets, systemic Ang II levels fall to values close to 10^−12^ mol/L [57]. Even though renal cortical Ang II levels during periods of excess salt intake are not known precisely, reductions in renal Ang II similar to plasma levels are expected to contribute to increase urinary Na^+^ excretion (UNaV) thereby eliminating excess salt. If UNaV fails to increase, there is a progressive positive balance of Na^+^ accompanied by retention of water and extracellular volume expansion. Together, these effects elevate arterial pressure. Blood pressure increases until pressure natriuresis achieves Na^+^ balance. Thus, if a primary reduction in Na^+^ reabsorption rates throughout the nephron fails to increase UNaV, Na^+^ balance would be achieved by elevating blood pressure leading to salt-sensitive hypertension [28,58].

Our data showing 20% fructose increases blood pressure are supported by previous studies using higher amounts of fructose in the diet. Several studies have shown that when 60% of caloric intake is made up by fructose that blood pressure increases even when rats are on a normal-salt base diet [59,60]. However, there is the caveat that these much greater amounts of fructose also cause metabolic syndrome, insulin resistance and increase plasma triglycerides [61]. Additionally, lower amounts of fructose cause hypertension over longer time frames of 8–12 weeks [62,63]. Taken together these results lead us to propose that the blood pressure effects of fructose are proportional to the amount of fructose, salt content of the diet, and time, such that when fructose makes up a high percentage of caloric intake it increases blood pressure in a short period of time even when animals are on normal salt. Conversely, low amounts of fructose only cause hypertension when the base diet contains excess salt over an extended period. The latter formulation may thus explain why some studies show no effect of fructose on blood pressure in human studies but others do, and the correlation of the increased incidence of hypertension with fructose and salt consumption in humans.

To test whether 20% fructose in the drinking water enhanced the sensitivity of the proximal tubule to Ang II when animals were on a high-salt base diet, we first measured QO_2_, which is an established screening method of rapidly measuring active Na^+^ transport in this segment. We found that 10^−12^ mol/L Ang II did not stimulate QO_2_ by proximal tubules from rats on high salt alone but did in tubules from rats consuming fructose plus high salt at one week suggesting that dietary fructose enhanced the sensitivity of the proximal nephron to this peptide. However, even though changes in QO_2_ by proximal tubules are a good indicator of changes in transport, several environmental factors can alter the efficiency of ATP production and oxygen utilization [64], and thereby could account for a part of the differences in QO_2_. In fact, we previously reported that dietary fructose affects the expression of several mitochondrial genes regardless of the salt content of the diet [55]. Thus, whenever alterations in QO_2_ were found, we further confirmed the results by direct assessment of transport rates in isolated tubules.

The first step in transcellular reabsorption of Na^+^ is the entry of the ion into the cell, which in proximal tubules largely depends on NHEs. To investigate whether changes in NHE activity was mediating the effects of fructose we assessed the activity of these transporters by pH recovery in isolated perfused tubules. We found that a low concentration of Ang II increased pH recovery in proximal tubules from HS-FRU rats but not in HS, in good agreement with the QO_2_ data. On the contrary, we found that dietary fructose did not alter the effects of low concentration of Ang II on Na^+^/K^+^-ATPase activity in neither HS-FRU nor HS tubules, when they were permeabilized and transmembrane Na^+^ gradients reduced. This indicates that the necessary increase in Na^+^/K^+^-ATPase activity to support elevated Na^+^ transport occurs secondary to enhanced apical Na^+^ entry and is not a direct effect of fructose feeding.

Proximal tubule fluid and bicarbonate reabsorption have a biphasic response to Ang II. Concentrations ranging from 10^−12^ to 10^−10^ mol/L increasingly stimulate transport, while concentrations of 10^−8^ mol/L and greater progressively reduce transport from the peak stimulated level to below baseline [30,31,65,66]. Our data show that 10^−12^ mol/L Ang II augmented both QO_2_ and NHE activity in tubules from rats consuming both fructose and high salt compared to high salt alone. The data also show that the augmentation of QO_2_ by 10^−9^ mol/L Ang II was similar in both groups. The results reported here are consistent with our previous report showing that 20% fructose in the drinking water elevated the sensitivity of proximal tubule Na^+^ reabsorption to Ang II at one week [38]. The simplest conclusion from these data is that fructose enhanced the sensitivity of proximal nephron transport to Ang II but did not alter the maximum rate.

ANP is a regulator of proximal tubule transport [44,67], and can be an important regulator of blood pressure. ANP knockout mice have hypertension and heterozygotes display salt-sensitive hypertension [68]. Decreasing ANP A receptor gene copy number causes salt-sensitivity while increasing it protects against hypertension [69]. Thus, a reduction in the inhibitory effects of ANP on proximal tubule Na^+^ reabsorption could contribute to fructose-induced salt-sensitive hypertension. As a result, we studied the ability of ANP to inhibit both QO_2_ and NHE activity in proximal tubules from HS and HS-FRU. We found that ANP reduced both parameters to the same extent in tubules from HS and HS-FRU. These data indicate that the inhibition of transport caused by ANP is not blunted by fructose-induced salt-sensitive hypertension nor the increase in Ang II sensitivity it causes.

This finding was somewhat surprising because ANP counteracts Ang II-stimulated fluid absorption in proximal tubules [70,71,72], and activation of protein kinase C by Ang II blunts the effects of ANP in other tissues [47,48]. The difference in our results and those from other tissues likely are due to differences in how ANP and Ang II interact. In proximal tubules the interaction involves cAMP [73] rather than protein kinase C and stimulation of cGMP phosphodiesterase activity as it does in glomeruli [47,48].

Finally, we tested whether the sensitivity to NE, another factor that stimulates Na^+^ reabsorption by proximal tubules, was enhanced by dietary fructose. The increase in blood pressure caused by dietary fructose has been shown to be blunted by acute administration of adrenergic antagonists [51], and the proximal tubule is richly innervated with sympathetic nerves. Furthermore, the signaling cascades activated by NE in this segment at least partially overlap with those stimulated by Ang II. However, our data show that the sensitivity to NE was not altered by dietary fructose on a high-salt base diet. These data indicate that the enhanced sensitivity to Ang II is not a generalizable effect. If the sensitivity to NE were enhanced, it would have indicated that the effects of fructose are not specific to Ang II.

In summary, consumption of a beverage containing 200 g/L of fructose for one week causes an increased sensitivity to Ang II, which allows proximal tubules to maintain high transport rates in the presence of low concentrations of the peptide. This, in combination with a high-salt diet leads to salt-sensitive hypertension.

## Figures and Tables

**Table 1:** Solutions and buffers.

	Bicarbonate-Buffered Physiological Saline	HEPES-Buffered Physiological Saline	K^+^-Free HEPES-Buffered Solution	Acid Pulse Buffer	4× Reaction Media	4× Reaction Media with Ouabain
	Concentrations in mmol/L					
NaHCO_3_	25.0	-	-	-	-	-
HEPES	-	10.0	10.0	10.0	-	-
Imidazole	-	-	-	-	200.0	200.0
NaCl	114.0	130.0	130.0	120.0	320.0	320.0
KCl	4.0	4.0	-	4.0	120.0	-
Na_2_HPO_4_	2.1	2.5	2.5	2.5	-	-
NaH_2_PO_4_	0.4	-	-	-	-	-
Mg SO_4_	1.2	1.2	1.2	1.2	20.0	20.0
Ca(Lactate)_2_	2.0	2.0	2.0	2.0	-	-
Na_3_Citrate	1.0	1.0	1.0	1.0	-	-
DL-alanine	6.0	6.0	6.0	6.0	-	-
Glucose	5.5	5.5	5.5	5.5	-	-
NH_4_Cl	-	-	-	10.0	-	-
EGTA	-	-	-	-	2.0	2.0
Na_2_ATP	-	-	-	-	20.0	20.0
NADH	-	-	-	-	4.0	4.0
Ascorbic Acid	-	-	-	-	4.0	4.0
PEP	-	-	-	-	40.0	40.0
Ouabain	-	-	-	-	-	1.0

HEPES: 4-(2-hydroxyethyl)-1-piperazineethanesulfonic acid. EGTA: ethylene glycol-bis (β-aminoethyl ether)-*N*,*N*,*N*′,*N*′-tetraacetic acid. NADH: nicotinamide adenine dinucleotide. PEP: phosphoenolpyruvate.

**Table 2:** Nutritional profile and growth.

		HS (*n* = 6)		HS-FRU (*n* = 6)		Change	*t*-Test
		Mean	SEM	Mean	SEM		
Beginning weight	(g)	163	7	160	8	=	*p* = 0.77
Final weight	(g)	222	12	202	13	=	*p* = 0.28
Weight gain	(g/24 h)	9	1	8	1	=	*p* = 0.43
Fluid intake	(mL/24 h)	64	3	46	2	↓	*p* < 0.01
Food intake	(g/24 h)	19	1	12	1	↓	*p* < 0.01
Caloric intake	(kcal/24 h)	74	3	80	5	=	*p* = 0.66
Na^+^ intake	(mEq/24 h)	14	1	9	1	*	

Rats fed a high-salt diet drinking tap water (HS) or 20% fructose (HS-FRU). “=” means “no change”, “↓” means “reduction”. * Values calculated from Food intake.

**Table 3:** Blood chemistry.

		HS (*n* = 6)		HS-FRU (*n* = 6)		Change	*t*-Test
		Mean	SEM	Mean	SEM		
Glucose	(mg/dL)	113	5	97	8	=	*p* = 0.12
Insulin	(pmol/L)	35	5	32	5	=	*p* = 0.69
pH		7.38	0.02	7.34	0.03	=	*p* = 0.21
Lactate	(mmol/L)	0.8	0.1	1.0	0.1	=	*p* = 0.25
Na^+^	(mmol/L)	137.7	0.5	138.2	0.4	=	*p* = 0.44
K^+^	(mmol/L)	3.9	0.1	3.9	0.1	=	*p* = 0.94
Cl^−^	(mmol/L)	109.3	0.6	108.6	0.6	=	*p* = 0.49

Rats fed a high-salt diet drinking tap water (HS) or 20% fructose (HS-FRU).
